# Draft Genome Sequence of *Leuconostoc mesenteroides* P45 Isolated from Pulque, a Traditional Mexican Alcoholic Fermented Beverage

**DOI:** 10.1128/genomeA.01130-14

**Published:** 2014-11-06

**Authors:** Fernando Riveros-Mckay, Itzia Campos, Martha Giles-Gómez, Francisco Bolívar, Adelfo Escalante

**Affiliations:** a Departamento de Ingeniería Celular y Biocátalisis, Instituto de Biotecnología, Universidad Nacional Autónoma de México (UNAM), Cuernavaca, Morelos, Mexico; b Winter Genomics, México DF, Mexico; c Departamento de Biología, Facultad de Química, UNAM, Ciudad Universitaria, Coyoacán, México DF, México

## Abstract

*Leuconostoc mesenteroides* P45 was isolated from the traditional Mexican pulque beverage. We report its draft genome sequence, assembled in 6 contigs consisting of 1,874,188 bp and no plasmids. Genome annotation predicted a total of 1,800 genes, 1,687 coding sequences, 52 pseudogenes, 9 rRNAs, 51 tRNAs, 1 noncoding RNA, and 44 frameshifted genes.

## GENOME ANNOUNCEMENT

Pulque is a traditional alcoholic, nondistilled fermented beverage produced by the fermentation of the sap known as aguamiel, extracted from native agave (maguey) species, mostly distributed in the states of the central Mexican plateau, where this beverage has been mainly produced and consumed (1). Studies on the microbiology of aguamiel and pulque showed the presence of a complex microbial diversity of bacteria and yeasts (2, 3), demonstrating that *Leuconostoc* species such as *L. citreum, L. kimchii*, and *L. mesenteroides* are among the most abundant lactic acid bacteria (LAB) present in the fresh sap and during the fermentation process (3). The genus *Leuconostoc* plays a key role in the fermentation of several traditional fermented products such as soured cereal doughs, vegetables, and beverages. The complete genome sequence of diverse *Leuconostoc* species isolated from these sources, including *L. mesenteroides, L. citreum, L. carnosum*, and *L. kimchii*, have been reported recently (4–7).

Consumption of pulque beverage has been traditionally associated with health-promoting effects (1). LAB involved in its fermentation have been proposed to possess probiotic properties (8, 9), highlighting the relevance of genome mining for genes coding for peptides and enzymes associated with antimicrobial activities in probiotic microorganisms (10).

*L. mesenteroides* strain P45 was isolated during a laboratory-controlled pulque fermentation, and chromosomal DNA was extracted as previously reported (3). The genome was sequenced at the University Massive DNA Sequence Unit, Instituto de Biotecnología, Universidad Nacional Autónoma de México (UUSMD-IBT, UNAM) in a Genome Analyzer IIx (Illumina) by multiplexing a paired-end and a mate-pair library.

The total sequence (2.68 Gbp, 1,432× coverage) was represented in 35,986,030 paired reads of 38 bp. Half of them were expected to contain the tag (paired end). Error correction and assembly were performed with SPAdes 3.1.0 (11). Contigs were ordered with Mauve (12) using the *L. mesenteroides* subsp. *mesenteroides* ATCC 8293 (NC_008531.1) genome as the reference-guide assembly. The draft genome of *L. mesenteroides* strain P45, which consists of 1,874,188 bp with an average GC content of 37.5%, was assembled into 6 contigs. Automatic genome annotation was performed using the Prokaryotic Genome Annotation Pipeline (13), which predicted a total of 1,800 genes, 1,687 coding sequences, 52 pseudogenes, 9 rRNAs (5S, 16S, 23S), 51 tRNAs, 1 noncoding RNA, and 44 frameshifted genes.

### Nucleotide sequence accession numbers.

This whole-genome shotgun project has been deposited at DDBJ/EMBL/GenBank under the accession number JRGZ00000000. The version described in this paper is version JRGZ01000000. The BioProject is PRJNA260860 and the BioSample is SAMN03032191.
